# Supplementary figures and images for: Self-organized patterning of peristaltic waves by suppressive actions in a developing gut
Source: Front Cell Dev Biol. 2025 Feb 27;13:1529975. doi: 10.3389/fcell.2025.1529975 (PMC11903454; doi:10.3389/fcell.2025.1529975)

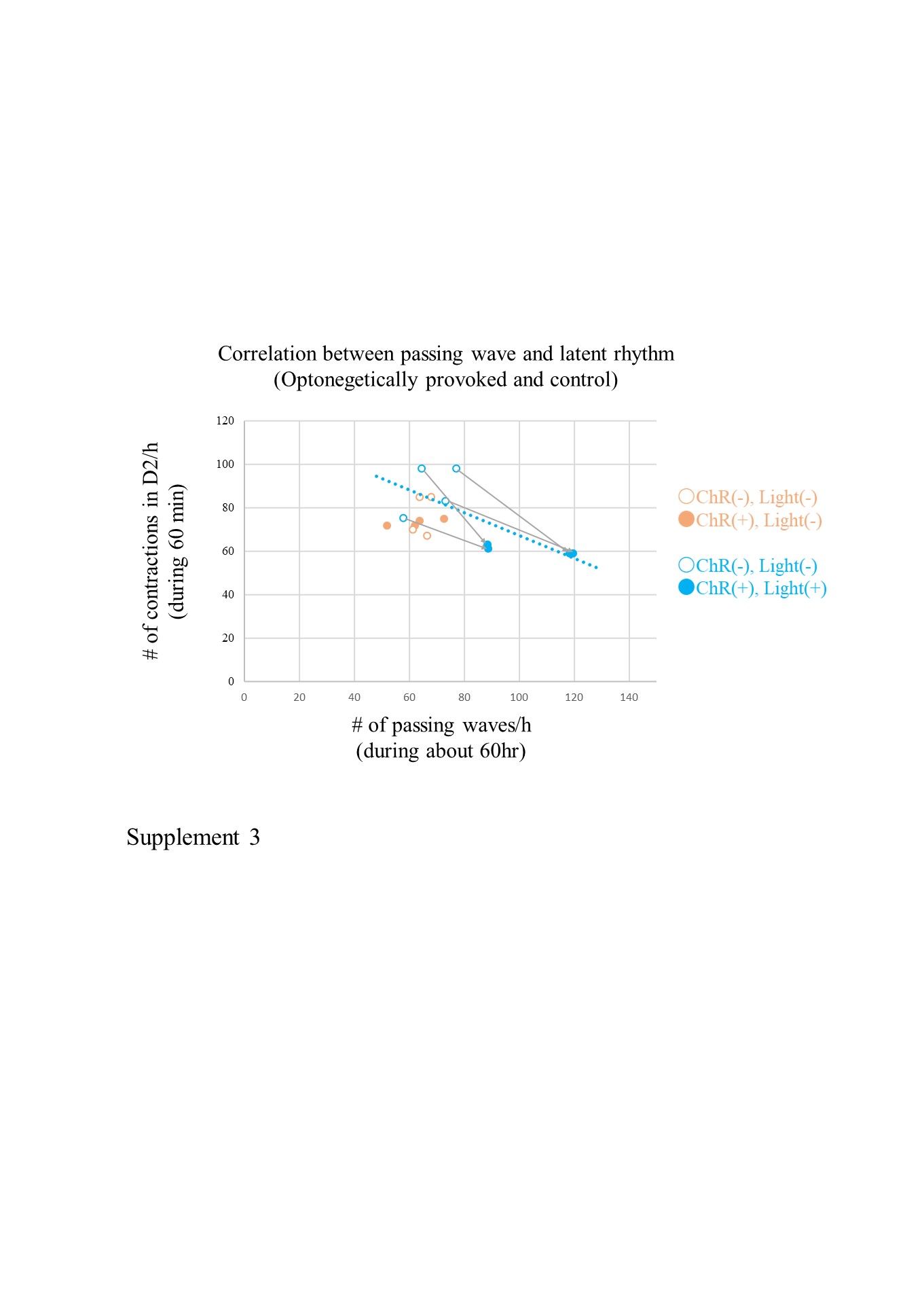

Supplement: Supplementary file 1 [file Image3.jpeg]

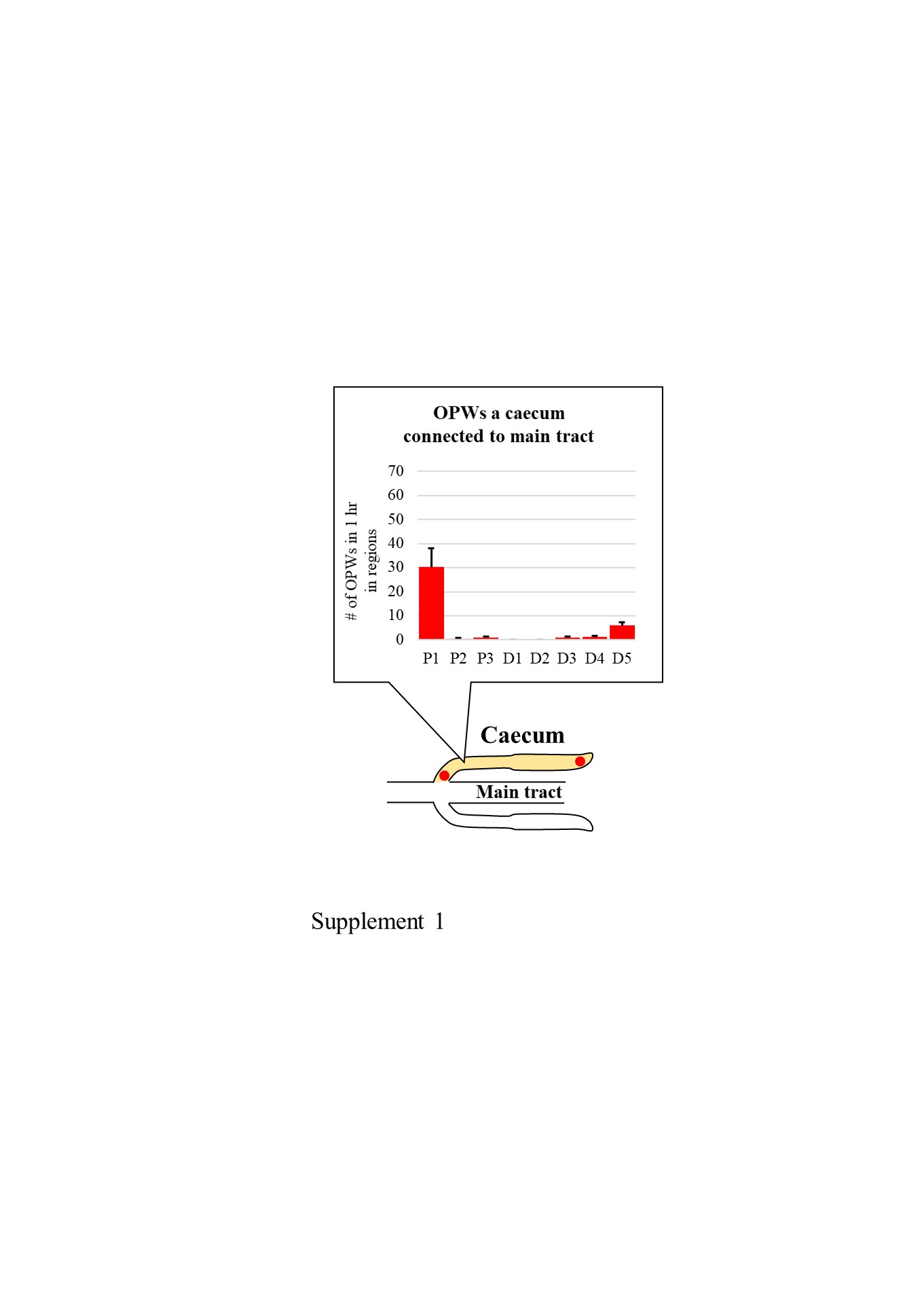

Supplement: Supplementary file 3 [file Image1.jpeg]

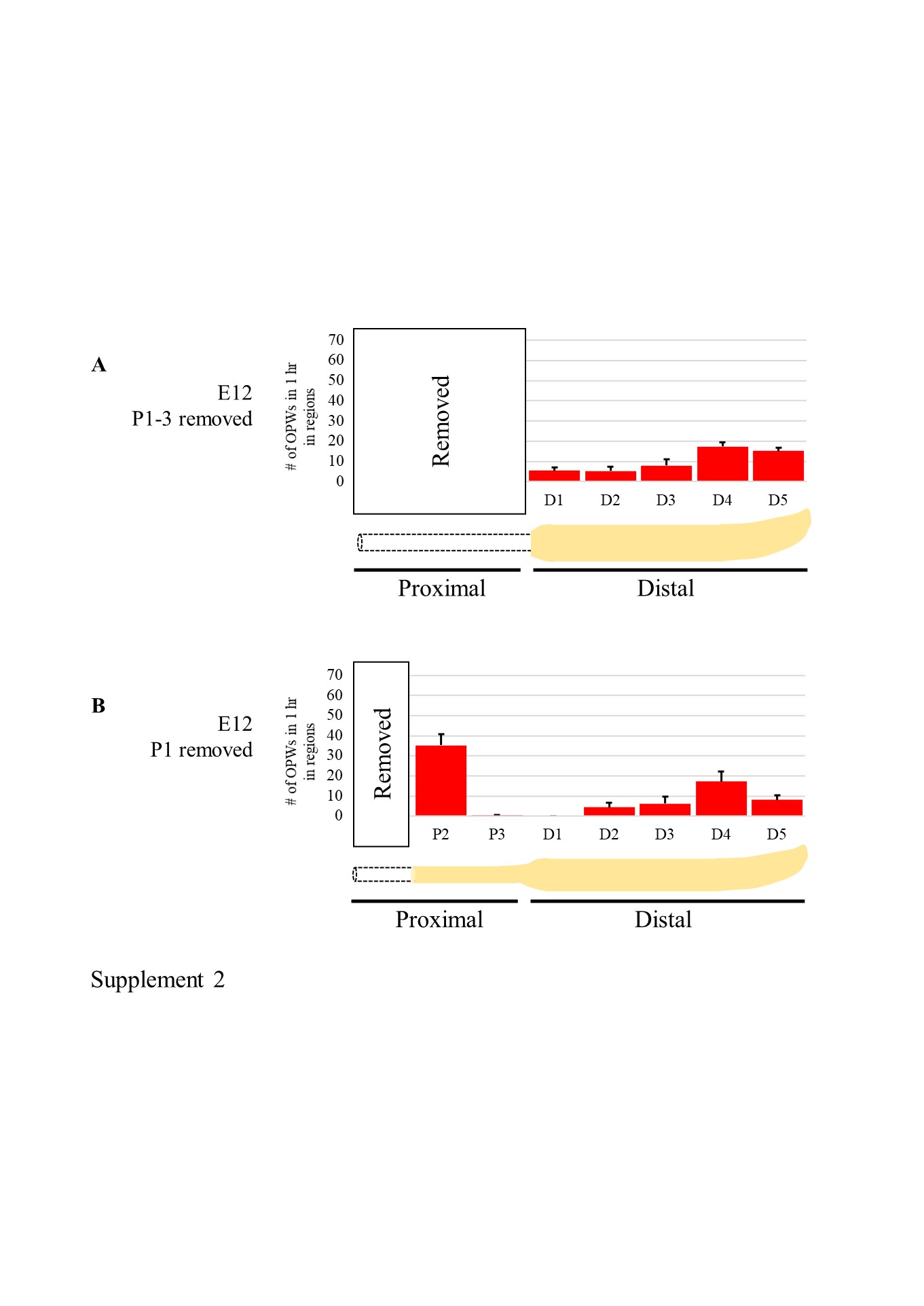

Supplement: Supplementary file 4 [file Image2.jpeg]
